# Use of Commercial Mixed-Mode Stationary Phases and Sorbents in the High-Performance Liquid Chromatography Analysis and Solid-Phase Extraction of Ionized and Hydrophilic Bioactive Compounds

**DOI:** 10.3390/molecules29102341

**Published:** 2024-05-16

**Authors:** Takeshi Fukushima, Mikoto Koishi, Tatsuya Sakamoto, Mayu Onozato

**Affiliations:** Department of Analytical Chemistry, Faculty of Pharmaceutical Sciences, Toho University, 2-2-1 Miyama, Funabashi-shi 274-8510, Chiba, Japan; 1019077k@st.toho-u.jp (M.K.); tatsuya.sakamoto@phar.toho-u.ac.jp (T.S.); mayu.onozato@phar.toho-u.ac.jp (M.O.)

**Keywords:** mixed-mode phase, high-performance liquid chromatography, solid-phase extraction, drug, pesticide, herbicide, serum, urine, environmental water

## Abstract

Mixed-mode high-performance liquid chromatography (HPLC) is increasingly used for the analysis of ionic and highly hydrophilic drugs, which are difficult to separate by conventional single-mode HPLC. In the former case, chromatographic separation is achieved using one of the several commercially available mixed-mode stationary phases, typically combinations of reversed and ion-exchange phases. Moreover, mixed-mode stationary phases can be used as solid-phase extraction (SPE) sorbents. This review focuses on the recent applications of mixed-mode stationary phases in the chromatographic analysis of bioactive compounds, such as drugs, herbicides, and pesticides. Specifically, we briefly summarize HPLC methods utilizing mixed-mode stationary phases and SPE pretreatment procedures utilizing mixed-mode sorbents developed in the last decade, thus providing a reference work for overcoming the difficulties in analyzing ionized or hydrophilic drugs by conventional reversed-phase chromatography.

## 1. Introduction

Traditional single-mode high-performance liquid chromatography (HPLC) most commonly exploits the reversed-phase retention mode, which largely corresponds to hydrophobic interactions between the analyte and stationary phase (e.g., octadecylsilyl group-modified silica) and is widely used for drug analysis. Given that the reversed-phase HPLC analysis of very hydrophilic or ionized drugs is hindered by their poor retention by the stationary phase, the hydrophobicity (and, hence, separation efficiency) of these analytes is typically increased using suitable hydrophobic ion-pairing agents. However, the introduction of ion-pairing agents into mobile phases generally reduces the detection sensitivity of mass spectrometry (MS), which is commonly applied in tandem with HPLC, although volatile ion-pairing agents, such as fluorinated alcohols and alkylamines, are used.

Unlike traditional HPLC, mixed-mode HPLC relies on two or more retention modes, typically the reversed-phase mode coupled with ion exchange and other modes, and is used in environmental research and pharmaceutical/illegal drug analysis [1,2] because of its applicability to ionized and hydrophilic analytes. To date, several types of mixed-mode stationary phases containing both hydrophobic and ion-exchange components have been developed and commercialized. The multimode interactions between charged analytes and mixed-mode stationary phases result in retention and separation patterns considerably different from those observed for solely hydrophobic or ion-exchange phases. In addition, the pH of the mobile phase is crucial for controlling the analyte charge and providing optimal separation conditions during HPLC.

Mixed-mode stationary phases have been classified into four groups (Types I–IV) by Zhang et al. [1,2]. Type I phases are physical mixtures of reversed (silica with grafted C18 alkyl chains) and ion-exchange (silica with grafted ionic groups) phases, while Type II phases comprise silica simultaneously grafted with reversed-phase and ion-exchange groups, and Type III and IV phases feature silica-bearing alkyl chains with chain-end or in-chain ionic groups.

The published works on mixed-mode HPLC analysis using stationary phases of Types I–IV include informative reviews and a book [1,2,3], focusing on several types of commercial mixed-mode columns. Moreover, mixed-mode phases have been used as sorbents for solid-phase extraction (SPE) pretreatment. Herein, we discuss the selected recent (2014–2024) applications (~20 papers) of commercial mixed-mode phases to the SPE and HPLC analysis of ionized and hydrophilic bioactive compounds, such as drugs, pesticides, and herbicides, to provide a reference for overcoming the difficulties in the related reversed-phase HPLC analysis.

## 2. Mixed-Mode Stationary Phases for the HPLC Analysis of Low-Molecular-Weight Bioactive Compounds

**Figure 1:**
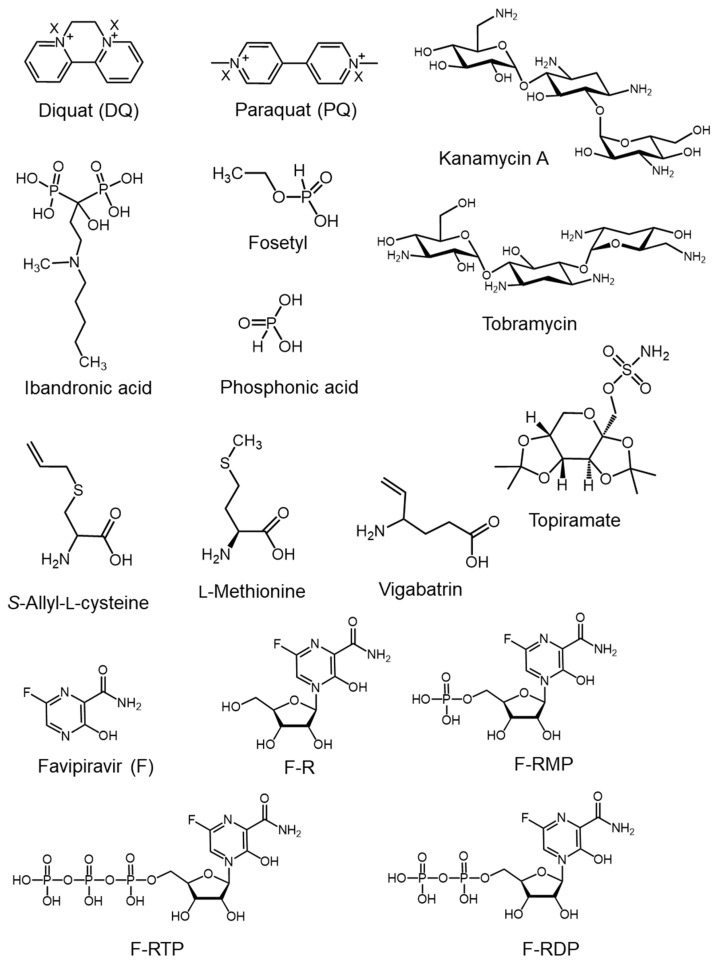
Chemical structures of bioactive compounds (drugs, herbicides, and pesticides) commonly analyzed by commercial mixed-mode high-performance liquid chromatography (HPLC).

The structures of the ionized and hydrophilic bioactive compounds commonly analyzed by mixed-mode stationary-phase HPLC and discussed herein are presented in Figure 1. Table 1 lists the corresponding analytical conditions, such as column name, mobile phase, and detection system.

### 2.1. Cationic Analytes

**Figure 2:**
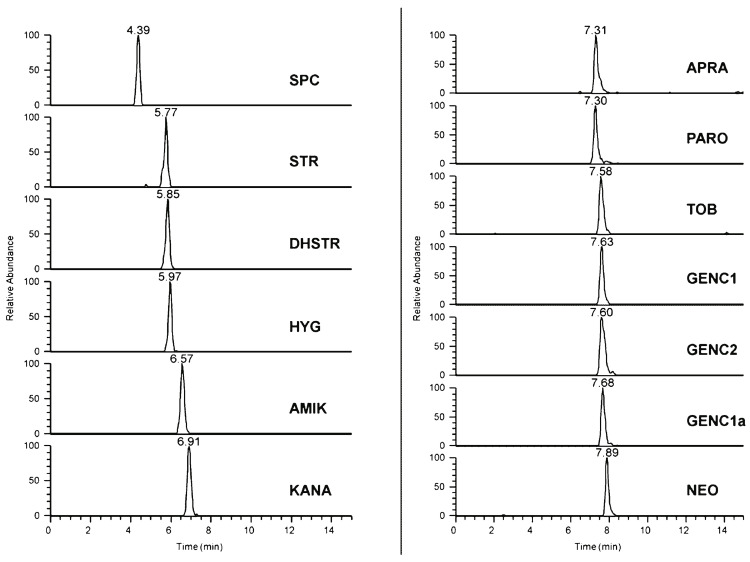
Chromatograms of a standard mixture of aminoglycosides. SPC: spectinomycin, STR: streptomycin, DHSTR: dihydrostreptomycin, HYG: hygromycin B, AMIK: amikacin, KANA: kanamycin A, APRA: apramycin, PARO: paromomycin, TOB: tobramycin, GENC1, GENC2, GENC1a: gentamicin, and NEO: neomycin. Reprinted with permission [4]. Copyright 2014 by Springer-Verlag Berlin Heidelberg.

Aminoglycosides (Figure 1), which contain several hydroxyl and amino groups and no hydrophobic moieties (e.g., long alkyl chains), are representative cationic drugs. In particular, kanamycin A, tobramycin, gentamicin, and neomycin are widely used broad-spectrum antibiotics, particularly in veterinary medicine. The high hydrophilicity of aminoglycosides hinders their separation by reversed-phase HPLC. The Obelisc R (150 × 2.1 mm, 5 μm) mixed-mode stationary phase column exhibiting three (reversed-phase, hydrophilic, and cationic) interactions was reported to effectively separate aminoglycosides via gradient elution with acetonitrile, water, and 1% formic acid in water (Table 1) [4]. TSQ Quantum Ultra AM triple quadrupole MS was used for quantification, and Q-Exactive^®^ high-resolution Orbitrap MS was used for accurate mass measurements. The liquid chromatography–tandem mass spectrometry (LC-MS/MS) system effectively separated and detected 13 types of aminoglycosides within 10 min (Figure 2) and enabled the determination of aminoglycosides in meat samples, such as veal, pork, and chicken [4]. As shown in Figure 2, the aminoglycoside peaks were sharp and did not show leading or tailing features.

Cationic herbicides, diquat (DQ) and paraquat (PQ) (Figure 1), were separated using the Acclaim^®^ Trinity Q1 column featuring reversed-phase, anion-exchange, and cation-exchange separation modes [5]. The target compounds of this study were 30 quaternary ammonium compounds extensively used as biocides, including benzalkyldimethylammonium compounds (BACs), alkyltrimethylammonium compounds, dialkyldimethylammonium compounds, hydroxy BACs, and carboxylic BACs. Among the quaternary ammonium compounds, structurally similar herbicides, DQ and PQ, which are both positively charged, were insufficiently separated by a reversed-phase column, even in the presence of heptafluorobutyric acid as an ion-pairing agent in the mobile phase. In contrast, baseline separation was achieved using a mixed-mode stationary phase column (Acclaim^®^ Trinity Q1) [5]. The authors proposed that cation-exchange interactions effectively separated DQ and PQ, while anion-exchange interactions could have minimized the undesirable analyte–silanol interactions. The optimal results were obtained for isocratic elution with 100 mM ammonium formate (pH 5.0):acetonitrile (25:75) (Table 1).

### 2.2. Anionic Analytes

Our literature survey of the HPLC analysis of anionic drugs indicated that mixed-mode stationary phases are frequently used to analyze phosphonate or bisphosphonate drugs because the phosphonate moiety is always negatively charged in aqueous media. Fosetyl, the ethyl ester of phosphonic acid (Figure 1), is a pesticide used to treat plant diseases. Fosetyl and phosphonic acid were separated using a mixed-mode anion-exchange column, Luna Omega PS C18 [7]. Although its stationary phase composition has not been disclosed by the manufacturer, this column effectively separated highly hydrophilic compounds, such as fosetyl and phosphonic acid, when a mobile phase comprising (A) 10% methanol in water and (B) 20 mM ammonium formate and 0.1% formic acid in water (pH 3.5) was used (Table 1). The sample injection volume was shown to be a critical factor. An injection volume of 1.0 μL provided a well-shaped phosphonic acid peak, whereas considerable peak fronting occurred at a higher injection volume of 5.0 μL.

Ibandronate (Figure 1) is a bisphosphonate drug used to treat osteoporosis. Given the negative charge on the bisphosphonate moieties and the positive charge on the tertiary amino group, the separation of ibandronate and related impurities by reversed-phase HPLC is challenging. However, ibandronate was successfully separated from its impurities using gradient elution with ultrapure water and 15% acetonitrile containing 15 mM trifluoroacetic acid on a column (Coresep^®^ SB) with a mixed-mode stationary phase bearing a strong cation-exchange group [8] (Table 1). This HPLC method was also suitable for other bisphosphonate drugs, such as etidronic and clodronic acids.

Favipiravir (T-705) (Figure 1) is a synthetic antiviral prodrug used to treat influenza [13,14], with the active form corresponding to favipiravir-ribofuranosyl-triphosphate (F-RTP). Some intermediate metabolites of favipiravir, such as favipiravir-ribofuranosyl-monophosphate (F-RMP), favipiravir-ribofuranose (F-R), and favipiravir-ribofuranosyl-diphosphate (F-RDP) (Figure 1), are produced enzymatically inside cells. Generally, phosphorylated metabolites are highly polar because of their negative charges.

Heart-cutting bidimensional LC using reversed-phase ACE3 C18-PFP and mixed-mode Primesep SB columns was used for therapeutic drug monitoring (Table 1) [6]. In the first separation by ACE3 C18-PFP, the phosphorylated metabolites were separated from F-R, F-M1, and the parent drug (favipiravir). In the next step, the mixture of phosphorylated metabolites (F-RMP, F-RDP, and F-RTP) was separated within 14 min by the mixed-mode Primesep SB column, and the elution order (F-RMP → F-RDP → F-RTP) suggested the dominance of anion-exchange interactions between the positively charged quaternary ammonium groups and negatively charged phosphate groups. The developed LC method was applied to biological samples, such as human serum, plasma, urine, and peripheral blood mononuclear cells. The lower limits of quantification, recoveries, and validation parameters were satisfactory.

### 2.3. Hydrophilic Analytes

Topiramate, an anticonvulsant (Figure 1), is highly soluble in water. For quality control applications, topiramate needs to be separated from its degradation products, such as fructose, sulfate, and sulfamide, which is challenging using reversed-phase HPLC. However, a mixed-mode phase column, Acclaim^®^ Trinity P1, enabled the effective separation of the inorganic and organic topiramate degradation products [9]. As these degradation products do not contain chromophores, a corona-charged aerosol detector (CAD) was used, as it is a universal detector with high sensitivity and a consistent response. The simultaneous separation of topiramate and its degradation products was achieved using isocratic elution with 20 mM ammonium acetate buffer (pH 4.0):methanol (80:20) (Table 1).

### 2.4. Amino Acids

Amino acids contain amino and carboxyl groups and are generally water-soluble. Therefore, these analytes are difficult to retain in the reversed-phase mode, especially when they have no aromatic or hydrophobic moieties in their side chains.

*S*-Allyl-L-cysteine (SAC), a biologically active component of garlic (*Allium sativum*), exhibits antioxidant, anticancer, antihepatopathic, and neurotrophic activities, and has therefore received attention as a health food supplement. The α-amino acid moiety of SAC complicates its retention during reversed-phase HPLC. However, SAC in rat plasma was successfully analyzed using a mixed-mode column (CAPCELL PAK CR 1:4) comprising a C18 reversed phase and strong cationic phase with sulfonate groups in a 1:4 ratio [10]. SAC was retained at 30–35 min in the mixed-mode phase with a mobile phase of 2 mM ammonium acetate (pH 3.5) and acetonitrile (75:25) (Table 1). This HPLC setup was successfully applied to a pharmacokinetic study in rats.

**Figure 3:**
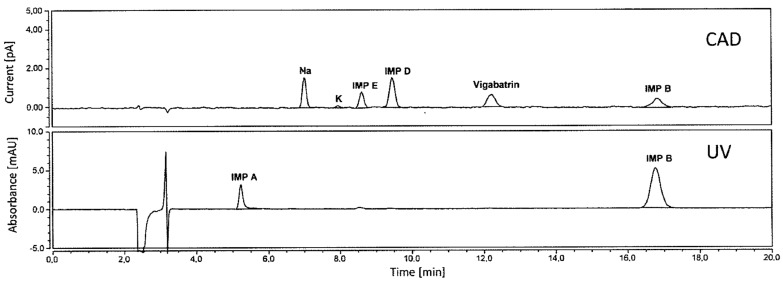
Chromatograms of a 0.03% solution of vigabatrin and its impurities. The upper and lower chromatograms correspond to CAD and ultraviolet detection, respectively. Reprinted with permission [11]. Copyright 2021, Elsevier B. V.

Similarly, mixed-mode stationary phases have been used for the impurity analysis of amino acid-type drugs. For example, the impurity analysis of vigabatrin (Figure 1), an anticonvulsant drug with *α*-amino acid moieties, was achieved using a mixed-mode stationary phase [11]. Vigabatrin and three types of impurities containing Na and K ions were effectively separated using a mixed-mode Primesep^®^ 100 column featuring acidic ion-pairing groups embedded into long hydrophobic ligand chains. The analysis time of 18.0 min obtained using water:acetonitrile (85:15) containing 0.1 vol% trifluoroacetic acid as the mobile phase (Table 1, Figure 3) was considerably lower than the value of ~30 min obtained using water:acetonitrile (85:15) containing 100 mM ammonium formate (pH 2.8). When a CAD system was used, Na and K ions accompanied with other impurities were detected simultaneously, whereas these impurity peaks were not detected by an ultraviolet detection system (Figure 3).

The synthesis of L-methionine can generate L-methionine sulfone, *N*-acetyl-L-methionine, *N*-acetyl-L-methionine-L-methionine, and *N*-acetyl-L-methionine-D-methionine as byproducts. The separation of these hydrophilic byproducts from each other and L-methionine is difficult to achieve using typical methods but was realized using a mixed-mode stationary phase column, Primesep^®^ 100 (Table 1) [12], which was also used for vigabatrin, as described above [11].

### 2.5. Chiral Low-Molecular-Weight Drugs and Biological Molecules

**Figure 4:**
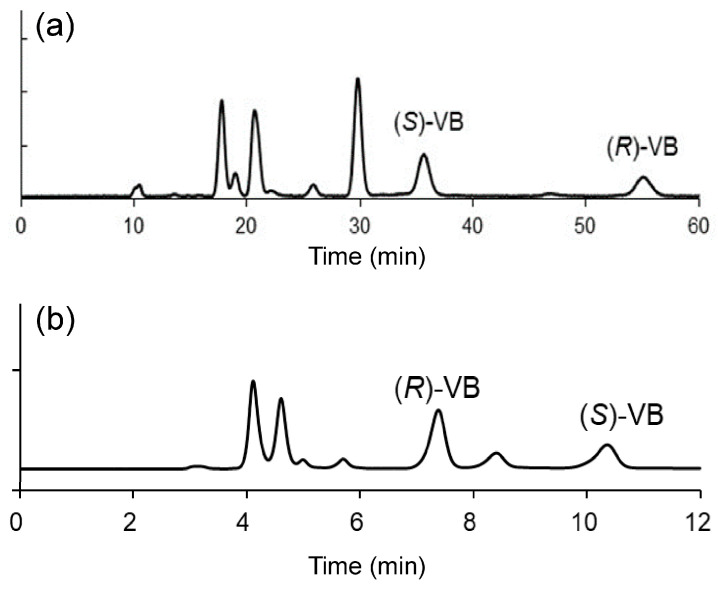
Chromatograms of Ns-MOK-(*R*)-Pro-OSu-derivatized racemic vigabatrin recorded using (**a**) a reversed-phase InertSustain^®^ C18 column and (**b**) a mixed-mode stationary-phase Scherzo^®^ SS-C18 column. Reprinted with permission. (**a**) Copyright 2020 by John Wiley & Sons, Ltd. [15]. (**b**) Copyright 2021 by Elsevier B. V. [16].

The chromatographic analysis of racemic drugs in biological fluids sometimes requires quantification data for each enantiomer because one enantiomer exhibits strong pharmacological activity or an undesirable side effect. Most current commercial mixed-mode phases contain no chiral moieties and are achiral. Therefore, precolumn diastereomer derivatization is necessary for the enantioseparation of chiral drugs or biocompounds by commercial achiral mixed-mode stationary phases. As mentioned above, the antiepileptic drug vigabatrin is a racemate. We previously reported that the precolumn derivatization of vigabatrin using a chiral fluorescent reagent, 2,5-dioxopyrrolidin-1-yl (4-[[(2-nitrophenyl)sulfonyl]oxy)-6-(3-oxomorpholino)quinoline-2-carbonyl)prolinate [Ns-MOK-(*R*)- or (*S*)-Pro-OSu], enables the enantiomers of vigabatrin to be effectively separated and fluorometrically detected using reversed-phase HPLC [15]. However, the analysis time considerably decreased when a mixed-mode stationary-phase column (Scherzo^®^ SS-C18) with a reversed phase and strong cation-/anion-exchange phases was used instead of a reversed octadecylsilyl phase (Figure 4) [16].

Furthermore, we performed precolumn diastereomer derivatization followed by separation using a mixed-mode stationary-phase column (Scherzo^®^ SS-C18) to analyze amino acids in commercial garlic foodstuffs [17]. The samples were derivatized with a chiral reagent, succinimidyl 2-(3-((benzyloxy)carbonyl)-1-methyl-5-oxoimidazolidin-4-yl)acetate (CIMa-OSu), and, consequently, several D- and L-amino acids were detected in black garlic samples.

**Figure 5:**
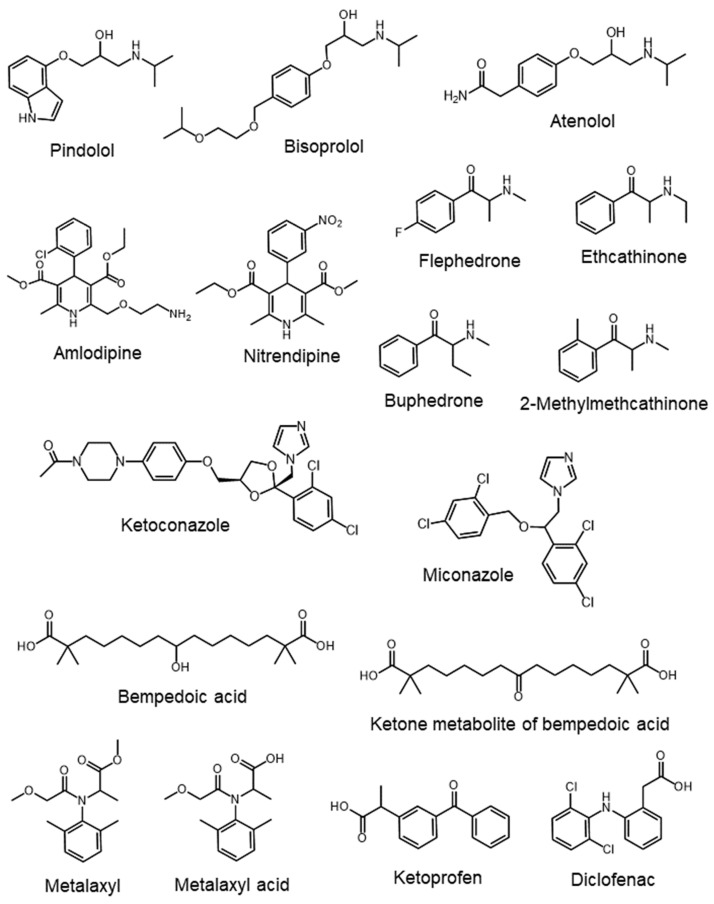
Chemical structures of basic and acidic drugs subjected to solid-phase extraction (SPE) pretreatment using a commercial or prepared cartridge packed with mixed-mode sorbents.

Novel mixed-mode stationary phases bearing chiral moieties have been reported. For example, a mixed-mode chiral stationary phase containing chiral hydroxyproline and octyl groups bound to silica particles enabled the effective separation of the enantiomers of a β-blocker, atenolol (Figure 5), within 20 min using methanol:0.2 mM copper sulfate (pH 4.6) (10:90) as the mobile phase [18].

## 3. Mixed-Mode Sorbents for SPE

Owing to its high selectivity and sensitivity, MS is the most common detection method, coupled with HPLC, for the analysis of biological and environmental samples. However, the interfering substances in the samples should be removed using an SPE purification step to avoid the degradation of the separation column and ion-suppression phenomenon in the MS detector. Below, we discuss prominent mixed-mode SPE sorbents used for the analysis of drugs with amino or carboxyl groups. The structures of these drugs are shown in Figure 5, and the SPE pretreatment procedures and conditions of the subsequent HPLC analysis are summarized in Table 2.

### 3.1. Basic Drugs

**Figure 6:**
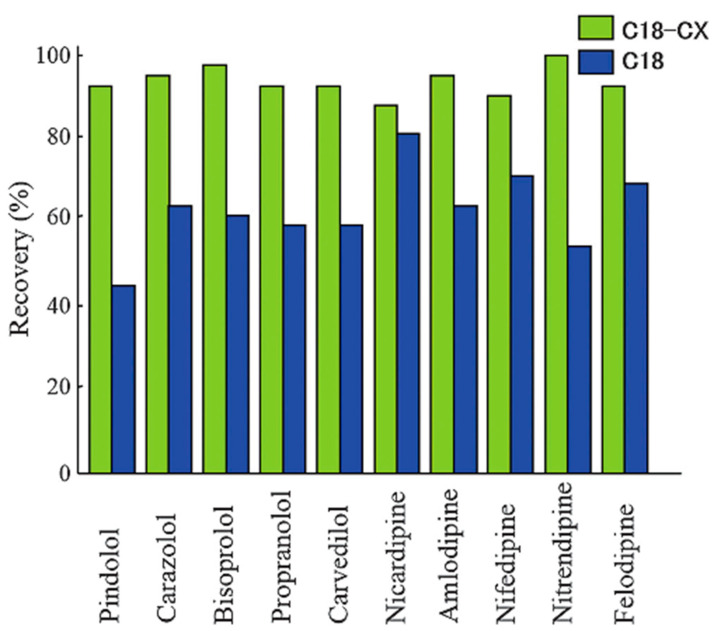
Recoveries of basic drugs subjected to SPE using reversed-phase (C18) and mixed-mode (C18-CX) sorbents. Reprinted from [19] under open access rules.

Some antihypertensive drugs, such as β-blockers (e.g., pindolol, bisoprolol, and carvedilol) and calcium-channel blockers (e.g., amlodipine and nitrendipine) (Figure 5), contain secondary amino groups and are therefore basic. Clinically, both of these drug types are often used together in combination therapy. Ten antihypertensive drugs in human serum were analyzed by LC-MS/MS after SPE pretreatment using a mixed-mode sorbent (MonoSpin C18-CX^®^) composed of a reversed phase and strong cation-exchange phase [19]. The cartridge was preactivated by elution with 0.5 mL of methanol and 0.5 mL of citrate buffer (10 mM, pH 3.0), loaded with the serum, citrate buffer (pH 3.0), and internal standard, and washed with the citrate buffer (pH 3.0). Methanol containing 2% NH_3_ (*v*/*v*) was used as the elution solvent, and neutralization with 1% HCl was necessary (Table 2). The recoveries of cardiovascular drugs achieved using the mixed-phase MonoSpin C18-CX^®^ column exceeded those achieved using the reversed-phase-only MonoSpin C18^®^ column (Figure 6). The authors noted that the pretreatment procedures are useful for the routine analysis of a large number of samples.

A mixed-mode cation-exchange sorbent, Oasis^®^ MCX, enabled the effective SPE of synthetic cathinones, β-keto phenethylamines, also known as bath salts, which are new psychoactive substances, in human urine [20]. Eleven synthetic cathinones, including flephedrone, ethcathinone, buphedrone, and 2-methylmethcathinone (Figure 5), were investigated. Given that synthetic cathinones contain secondary amino groups and are therefore basic (p*K*_a_ = 7.3–8.2), a phosphate buffer solution adjusted to pH 6.0 was mixed with 5 mL of urine (50:50) to ensure cationic interactions between the sorbent and cathinones. The urine sample mixed with the phosphate buffer (pH 6.0) was loaded into a 150 mg Oasis MCX cartridge, washed with 2 mL of methanol, and eluted with 2 mL of 5% NH_4_OH in methanol (Table 2). In view of the volatility of the examined cathinones, the final solution was supplemented with 100 μL of 1% HCl in methanol to avoid evaporation losses. The recoveries from the SPE cartridge ranged from 84% to 101% (Table 2). In addition, the matrix effect of MS detection was reduced when the Oasis WCX cartridge was replaced with the Oasis MCX cartridge.

Antimycotic drugs released into environmental water can affect aquatic organisms. A representative study demonstrated the LC-MS determination of antimycotic drugs, such as ketoconazole, miconazole, fluconazole, econazole, clotrimazole, and etaconazole (Figure 5), in environmental water, effluent of sewage treatment plants, and river water [21]. Given that antimycotic drugs mostly feature weakly basic groups, such as imidazole and triazole moieties, their SPE extraction was achieved using an Oasis^®^ MCX column with reversed-phase and cationic-exchange sorbents. After conditioning with 5 mL of methanol and 5 mL of ultrapure water (adjusted to the same pH as the water samples), the column was washed with 5 mL of methanol:water (10:90) and 2.5 mL of 0.1% formic acid in methanol, and eluted with 2 mL of methanol containing 2% NH_3_ (*v*/*v*). The recoveries were 84–104% (river water), 71–109% (treated water), and 72–92% (raw wastewater) (Table 2). This pretreatment reduced the MS matrix effect and thus facilitated subsequent analysis by LC–time-of-flight MS.

### 3.2. Acidic Drugs

Bempedoic acid (Figure 5) is a potent inhibitor of ATP-citrate lyase and holds promise for the treatment of hypercholesterolemia. Given that bempedoic acid has two carboxyl groups, an SPE procedure with a mixed-mode phase (Oasis^®^ MAX; 30 mg/well, 30 μm) was used to extract this acid and its keto metabolites (Figure 5) from human urine [23]. Preconditioning was performed using 1.0 mL of water:methanol (5:95) and 1.0 mL of 100 mM ammonium formate (pH 3.8). After the loading of the urine sample, the column was washed with 100 mM ammonium formate (pH 3.8) and water, and eluted with 800 μL of formic acid:ethanol (2:98) (Table 2). Prior to being loaded into the SPE column, the human urine was acidified with 85% phosphoric acid (99:1) and supplemented with 25% isopropyl alcohol to avoid glucuronide hydrolysis and nonspecific binding to vessel walls.

Metalaxyl (Figure 5) is a fungicide used to treat fungal infections and improve food safety. Given that the main metabolite of this fungicide is metalaxyl acid (Figure 5), the simultaneous SPE pretreatment of metalaxyl and metalaxyl acid enantiomers in animal-derived food samples, such as beef, pork, chicken, and fish, was performed using an Oasis^®^ MAX cartridge [22]. The specific SPE conditions were optimized. The cartridge was preconditioned using 3 mL of methanol, 3 mL of water, and 3 mL of 0.2% aqueous ammonia. After food sample loading, the cartridge was washed with 3 mL of 20% aqueous methanol and 3 mL of 0.2% aqueous ammonia, and eluted with 3 mL of 0.5% formic acid in methanol (Table 2). Following SPE, a chiral stationary phase, EnantioPak^®^ Y1-R, was used for the quantification of metalaxyl and metalaxyl acid enantiomers, with recoveries ranging from 89.5% to 110.3% (Table 2).

Nonsteroidal anti-inflammatory drugs (NSAIDs), such as ibuprofen, flurbiprofen, and ketoprofen, are clinically important drugs containing carboxyl groups. A hyperbranched mixed-mode anion-exchange polymeric SPE sorbent with quaternary ammonium moieties was prepared by sequentially reacting aminated poly(divinylbenzene) with resorcinol diglycidyl ether (80 °C, 3 h) and methylamine (80 °C, 3 h) [24]. Five types of quaternary hyperbranched macromolecules were prepared as mixed-mode anion-exchange (MAX) sorbents and packed into a 3 mL polypropylene SPE tube for testing. The prepared cartridge was conditioned with 5 mL of methanol and 5 mL of 10 mM phosphate buffer (pH 2.0). After loading human urine samples treated by alkali hydrolysis (~7 mL), the samples were consecutively washed with 3 mL of 15% acetic acid and 5 mL of 5% methanol in 50 mM sodium acetate. After the removal of residual water under vacuum for 30 min, elution experiments were performed with 1–4 mL of 1% formic acid in methanol for 50, 100, and 200 mg of the sorbent. The recoveries achieved for nine types of NSAIDs in human urine were acceptable and similar to those obtained using a commercial SPE sorbent, Oasis MAX (Table 2). In addition, the NSAIDs were separated from neutral and basic drugs (e.g., carbamazepine, hydrocortisone, and amitriptyline) with similar log *p* values based on differences in their SPE elution profiles.

## 4. Conclusions and Future Directions

Our survey of recently published papers shows that mixed-mode stationary phases are increasingly used for the analysis of hydrophilic drugs, herbicides, and pesticides (along with their metabolites and impurities) in biological and food samples.

As shown in Table 1, optimizing the pH of the mobile phase is crucial for achieving optimal separation, as the ionic form of the target drug is dominant during ion-exchange interactions with the mixed-mode stationary phase in addition to hydrophobic interactions with the reversed phase. Therefore, one should aim to obtain data on the p*K*_a_ of the target drugs for the best separation before using the mixed-mode stationary phase.

Analytes separated by the mixed-mode stationary phase are typically detected using MS/MS or CAD systems, which can detect compounds without suitable chromophores, including highly polar compounds (e.g., phosphonic acid).

Mixed-mode stationary phases have also been used for purifying medium-size compounds, such as synthetic oligonucleotides, which have numerous anionic phosphate linkages [25]. Chemically modified oligonucleotides have been developed as therapeutics. Although synthetic oligonucleotides are typically analyzed by reversed-phase HPLC using volatile ion-pairing agent-supplemented mobile phases [26], a mixed-mode stationary phase (Scherzo^®^ SS-C18) was found to be useful for the analysis of *N*-acetylgalactosamine-modified small interfering ribonucleic acids [25] and ionized medium-size compounds, such as oligonucleotides.

Additionally, mixed-mode sorbents are beneficial for SPE pretreatment during the analysis of ionized or hydrophilic drugs, helping to overcome the limitations of reversed-phase sorbents. In the case of mixed-mode sorbents for SPE, methanol is more commonly used as an organic component of the eluent than acetonitrile (Table 2). In addition, the use of a mixed-mode SPE sorbent can minimize matrix effects during subsequent MS detection. Thus, mixed-mode stationary phases and sorbents are expected to contribute to clinical, medicinal, and environmental research.

## Figures and Tables

**Table 1:** Conditions and LOD/LOQ of the mixed-mode HPLC analysis of selected bioactive compounds.

Analyte	Mixed-Mode Column	Mobile Phase	Detection	LOD/LOQ	Sample	Ref.
Cationic drugs and herbicides						
Aminoglycoside antibiotics	Obelisc^®^ R column (SIELC) (150 × 2.1 mm, 5 μm)	Gradient elution with A: acetonitrile, B: water, C: 1% formic acid in water	MS	Spectinomycin: 3 pg (LOD) Gentamicin C2: 30 pg (LOD)	Minced meat (veal, pork, and chicken)	[4]
Diquat, paraquat	Acclaim^TM^ Trinity Q1 (Thermo Fisher Scientific) (100 × 2.1 mm, 3 µm)	Isocratic elution (A:B = 25:75) with A: 100 mM ammonium formate (pH 5.0), B: acetonitrile	MS	Diquat: 0.04 ng/mL (LOD) Paraquat: 0.05 ng/mL (LOD)	Human serum and urine	[5]
Anionic drugs and impurities						
Favipiravir-RMP, RDP, RTP	Primesep SB column (SIELC) (150 × 4.6 mm, 5 μm)	Isocratic elution (A:B = 99:1) with A: 180 mM ammonium acetate (pH 4.6), B: acetonitrile	DAD	Favipiravir: 0.4 mg/L (LLOQ; serum, plasma, PBMCs) Favipiravir-ribofuranose: 10 mg/L (LLOQ, PBMCs) Favipiravir-ribofuranosyl-monophosphate: 16 mg/L (LLOQ, PBMCs) Favipiravir-ribofuranosyl-diphosphate: 20 mg/L (LLOQ, PBMCs) Favipiravir-ribofuranosyl-triphosphate: 4 mg/L (LLOQ, PBMCs) 5-hydroxyfavipiravir: 5.0 mg/L (LLOQ, urine)	Human serum, plasma, urine, and PBMCs	[6]
Fosetyl and phosphonic acid	Luna Omega PS C18 (Phenomenex) (100 mm × 3 mm, 3 μm)	Gradient elution with A: 10% methanol in water B: 20 mM ammonium formate and 0.1% formic acid in water (pH 3.5)	MS	Fosetyl: 0.02 mg/kg (LOQ, spinach), 0.02 mg/kg (LOQ, cherry), 0.20 mg/kg (LOQ, wheat flour) Phosphonic acid: 0.02 mg/kg in spinach, 0.02 mg/kg in cherry, 0.20 mg/kg in wheat flour (LOQ)	Food (spinach, cherry, arugula, lettuce, wheat, and oat flour)	[7]
Ibandronate sodium and its impurities	Coresep^®^ SB (SIELC) (150 × 4.6 mm, 2.7 μm)	Gradient elution with A: ultrapure water, B: 15% acetonitrile containing 15 mM trifluoroacetic acid	CAD MS	*N*-Pentyl-*N*-methyl-β-alanine: 0.03% (LOQ) Phosphate: 0.02% (LOQ) Despentylibandronate: 0.02% (LOQ) Olpadronate: 0.03% (LOQ) Phosphite: 0.02% (LOQ) Desmethylibandronate: 0.02% (LOQ)	Batch test	[8]
Hydrophilic drugs and impurities						
Topiramate and its main degradation products	Acclaim^TM^ Trinity P1 (Dionex) (150 × 3.0 mm, 2.7 μm)	Isocratic elution (A:B = 80:20) with A: 20 mM ammonium acetate buffer (pH 4.0), B: methanol	CAD	Topiramate: 2.97 μg/mL (LOD), 11.15 μg/mL (LOQ) Fructose: 12.08 μg/mL (LOD), 40.28 μg/mL (LOQ) Sulfate: 4.02 μg/mL (LOD), 13.41 μg/mL (LOQ) Sulfamate: 13.91 μg/mL (LOD), 46.38 μg/mL (LOQ) Compound A: 3.94 μg/mL (LOD), 13.13 μg/mL (LOQ)	Standard	[9]
Amino acids and their impurities						
S-Allyl-L-cysteine	CAPCELL PAK CR 1:4 (Shiseido) (100 × 2.0 mm, 3 μm)	Isocratic elution with A: 2 mM ammonium acetate buffer (pH 3.5), B: 0.1% formic acid in acetonitrile	MS	*S*-Allyl-L-cysteine: 5 ng/mL (LOQ)	Rat plasma	[10]
Vigabatrin impurities	Primesep^®^ 100 (SIELC) (250 × 4.6 mm, 5 μm)	Isocratic elution with water/acetonitrile (85:15) containing 0.1 vol% trifluoroacetic acid	CAD	Impurity A: 9 ng (0.006%) Impurity B: 6 ng (0.004%) Impurity D: 12 ng (0.008%) Impurity E: 18 ng (0.012%)	Standard	[11]
L-Methionine impurities	Primesep^®^ 100 (SIELC) (250 × 4.6 mm, 5 μm)	Isocratic elution (A:B = 80:20) with A: 12.5 mM aqueous phosphoric acid, B: acetonitrile	Ultraviolet (210 nm)	AcMet: 0.06 μg/mL (LOD), 0.30 μg/mL (LOQ) AcMetMet 1: 0.06 μg/mL (LOD), 0.30 μg/mL (LOQ) AcMetMet 2: 0.06 μg/mL (LOD), 0.30 μg/mL (LOQ) MetOx: 0.30 μg/mL (LOD), 0.75 μg/mL (LOQ)	Standard	[12]

AcMet: *N*-Acetyl-DL-methionine; AcMetMet 1: *N*-Acetyl-L-methionine-L-methionine and enantiomer; AcMetMet 2: *N*-Acetyl-L-methionine-D-methionine and enantiomer; CAD: corona-charged aerosol detection; DAD: diode array detector; LOD: limit of detection; LLOQ: lower limit of quantification; LOQ: limit of quantification; MetOx: L-methionine sulfoxide; MS: mass spectrometry; PBMCs: peripheral blood mononuclear cells. Here and in other cases, *v*/*v* solvent ratios are meant unless stated otherwise.

**Table 2:** Mixed-mode sorbents for the SPE pretreatment of basic and acidic drugs.

Analyte	SPE Process				Chromatography					
	SPE Sorbent	Preconditioning	Washing	Elution	Column	Mobile Phase	Detection	Sample Matrix	Recovery	Ref.
Basic drugs										
Cardiovascular drugs	MonoSpin C18-CX^®^	0.5 mL of methanol, 0.5 mL of citrate buffer (pH 3)	0.4 mL of citrate buffer (pH 3)	0.1 mL of 2% NH_3_ in methanol	InertSustain^®^ C8 (150 × 2.1 mm, 3 μm)	Elution with A: 10 mM ammonium formate containing 0.1% formic acid (pH 3.3) B: acetonitrile or methanol	MS	Human serum	76–108%	[19]
Synthetic cathinones (*β*-keto phenethylamines)	Oasis^®^ MCX (150 mg/6 mL)	5 mL of methanol, 5 mL of phosphate buffer solution (pH 6)	2 mL of methanol	2 mL of 5% NH_4_OH in methanol	Luna Omega 5 μm Polar C18 (150 × 4.6 mm, 5 μm)	Gradient elution with A: 0.1% formic acid in ultrapure water B: 0.1% formic acid in acetonitrile	High-resolution MS	Human urine	84–101%	[20]
Antimycotic drugs	Oasis^®^ MCX (150 mg)	5 mL of methanol, 5 mL of ultrapure water, adjusted to the same pH as water samples	5 mL of methanol:water (10:90) 2.5 mL of methanol (0.1% formic acid)	2 mL of methanol containing 2% NH_3_ (*v*/*v*)	Zorbax Eclipse XDB C18 (100 × 2 mm, 3.5 μm)	Gradient elution with A: 5 mM ammonium acetate in ultrapure water B: 5 mM ammonium acetate in methanol	MS	Environmental water (sewage treatment plants, river water)	84–104% (river water) 71–109% (treated water) 72–92% (raw wastewater)	[21]
Acidic drugs or metabolites										
Metalaxyl, metalaxyl acid	Oasis^®^ MAX (60 mg, 3 mL)	3 mL of methanol, 3 mL of water, 3 mL of 0.2% aqueous ammonia	3 mL of 20% aqueous methanol, 3 mL of 0.2% aqueous ammonia	3 mL of 0.5% formic acid in methanol	EnantioPak^®^ Y1R (150 × 4.6 mm, 5 μm)	Acetonitrile:H_2_O:formic acid (60:40:0.1)	MS/MS	Muscle tissues (veal, pork, chicken, fish)	89.5–110.3%	[22]
Bempedoic acid, ketone metabolite	Oasis^®^ MAX (30 mg/well, 30 μm)	1.0 mL of water:methanol (5:95), 1.0 mL of 100 mM ammonium formate buffer (pH 3.8)	1.0 mL of 100 mM ammonium formate buffer (pH 3.8), 1.0 mL of water	800 μL of formic acid:ethanol (2:98)	BEH C18 (50 × 2.1 mm, 1.7 μm)	Gradient elution with A: water:methanol:formic acid (90:10:0.1) B: water:methanol:formic acid (10:90:0.1)	MS/MS	Human urine	88.2–96.9% (bempedoic acid), 89.9–94.1% (ketone metabolite)	[23]
NSAIDs	G4-QHMs MAX sorbent	5 mL of methanol, 5 mL of 10 mM phosphate buffer (pH 2.0)	3 mL of 15% acetic acid, 5 mL of 5% methanol in 50 mM sodium acetate (pH 7) 5 mL of methanol	4 mL of 1% formic acid in methanol	Agilent Zorbax SB-C18 (250 × 4.6 mm, 5 μm)	Gradient elution with A: 20 mM KH_2_PO_4_ (pH 2.7); B: methanol Isocratic elution for ibuprofen; 20 mM KH_2_PO_4_ (pH 2.7):methanol (20:80)	Ultraviolet	Human urine	81.9–104.0%	[24]

## Data Availability

No new data were created or analyzed in this study. Data sharing is not applicable to this article.
